# Reply: Mobile phone use and acoustic neuroma in five North European countries

**DOI:** 10.1038/sj.bjc.6603071

**Published:** 2006-03-28

**Authors:** M J Schoemaker, A J Swerdlow, A Auvinen, E Cardis, M Feychting, C Johansen, P A McKinney, T Tynes

**Affiliations:** 1 Section of Epidemiology, Institute of Cancer Research, Sutton SM2 5NG, UK; 2 STUK-Radiation and Nuclear Safety Authority, 00881, Helsinki, Finland; 3 Tampere School of Public Health, University of Tampere, Tampere, 33014, Finland; 4 International Agency for Research on Cancer, 150 Cours Albert Thomas, 69372 Cedex 08, Lyon, France; 5 Institute of Environmental Medicine, Karolinska Institute, Box 210, 171 77, Stockholm, Sweden; 6 Institute of Cancer Epidemiology, Danish Cancer Society, Strandboulevarden 49, 2100 Copenhagen, Denmark; 7 Centre for Epidemiology and Biostatistics, University of Leeds, 30 Hyde Terrace, Leeds LS2 9LN, UK; 8 The Cancer Registry of Norway, Institute of Population-based Cancer Research, Montebello, 0310, Oslo, Norway; 9 The Norwegian Radiation Protection Authority, PO Box 55, 1332 Osteras, Norway

**Sir**,

We thank the correspondents for their interest in our study (Schoemaker *et al*, 2005). Professors Hardell and Mild ask about several design features of the study, to which we reply as follows.

Controls in the four Nordic centres in our study were recruited from population registers and from the UK centres were selected from general practitioner practices. The latter are a representative source of population-based controls as approximately 98% of the UK population is registered with a GP (OPCS, 1992).

With exception of Finland, where interviews were nearly all at a hospital, interviews were carried out at a location chosen by the participant, with interviewers travelling to these locations, and at a time of interview most convenient to the participant, to make the interview as stress-free as possible. Blinding interviewers to participants' case–controls status is impossible in personal interviews, but interviewers were trained to treat everyone equally and information on mobile phone use was collected with a highly structured and standardized computer-assisted interview. Exposure indices were calculated with computer programs regardless of case–control status. Excluding the small proportion of subjects who were interviewed over the telephone, mainly in Norway, did not materially affect the results. Contrary to the correspondents' assertion, the Northern part of Sweden was not part of the study region of the Swedish study. Patients or controls at distant addresses in the study were not omitted from the study.

With regard to Table 4 in our paper, we refer Professors Hardell and Mild to the Methods section and the table's footnote describing the definition of the reference groups and the inclusion of bilateral phone users in both the ipsilateral and contralateral analyses.

We did not include cordless phones in our analysis because they have a lower power output than mobile phones (IEGMP, 2000), but several other Interphone papers did include them (e.g. Lonn *et al*, 2004; Schüz *et al*, 2006), and the pooled 13-country Interphone analyses will be able to explore whether the exclusion of cordless phones makes a material difference to the results.

Provision of funds to the study investigators was via a firewall and was governed by legally binding agreements that guaranteed the study's complete scientific independence. The research was initiated, conducted and published without reference to any of the funding agencies. It is hard to see how a contractual clause that the funders may be informed a maximum of 7 days before publication of the results under strict terms of confidentiality infringes on this.

Dr Hocking points out that misclassification in exposure assessment could have diluted a possible increased risk of acoustic neuroma among mobile phone users. We explain, in the paper, that misclassification could have diluted any real trend of risk with cumulative number of calls or hours of use, but as Dr Hocking points out himself, information on recall of year of first phone use is less likely to be seriously erroneous. Part of the reason for our conclusion of ‘no substantial risk in the first decade after starting mobile phone use’ was exactly this information.

Dr Milham focuses on the decreased odds ratios in relation to recent phone use, and the possibility of control selection bias. We had discussed in our paper the potential for this bias, but also the potential for other biases, some of which might explain the decreased relative risks after recent use. As there is more than one explanation for a diminished relative risk after short-term use, and uncertainty on the extent and presence of the various biases affecting the relative risk, we do not think that one can justifiably draw any conclusions about a ‘future epidemic’, but rather, as we stated, that ‘an increase in risk after longer-term use or after a longer lag period could not be ruled out’.
